# Medicinal Plants: A Public Resource for Metabolomics and Hypothesis Development

**DOI:** 10.3390/metabo2041031

**Published:** 2012-11-21

**Authors:** Eve Syrkin Wurtele, Joe Chappell, A. Daniel Jones, Mary Dawn Celiz, Nick Ransom, Manhoi Hur, Ludmila Rizshsky, Matthew Crispin, Philip Dixon, Jia Liu, Mark P. Widrlechner, Basil J. Nikolau

**Affiliations:** 1 Department of Genetics, Cell and Developmental Biology, Iowa State University, Ames, IA 50011, USA; 2 Center for Metabolic Biology, The Plant Science Institute, Iowa State University, Ames, IA 50011, USA; 3 Department of Cellular and Molecular Biochemistry, University of Kentucky, Lexington, KY, 40536, USA; 4 Department of Biochemistry & Molecular Biology and Deptment of Chemistry, Michigan State University, East Lansing, MI 48824, USA; 5 Department of Biochemistry, Biophysics and Molecular Biology, Iowa State University, Ames, IA 50011, USA; 6 Department of Statistics, Iowa State University, Ames, IA 50011, USA; 7 Department of Ecology, Evolution, and Organismal Biology and Department of Horticulture, Iowa State University, Ames, IA 50011, USA

**Keywords:** database, metabolomics, specialized metabolites, medicinal, cardiac glycoside, alkaloid, digitalis, terpene, phenolic.

## Abstract

Specialized compounds from photosynthetic organisms serve as rich resources for drug development. From aspirin to atropine, plant-derived natural products have had a profound impact on human health. Technological advances provide new opportunities to access these natural products in a metabolic context. Here, we describe a database and platform for storing, visualizing and statistically analyzing metabolomics data from fourteen medicinal plant species. The metabolomes and associated transcriptomes (RNAseq) for each plant species, gathered from up to twenty tissue/organ samples that have experienced varied growth conditions and developmental histories, were analyzed in parallel. Three case studies illustrate different ways that the data can be integrally used to generate testable hypotheses concerning the biochemistry, phylogeny and natural product diversity of medicinal plants. Deep metabolomics analysis of *Camptotheca acuminata* exemplifies how such data can be used to inform metabolic understanding of natural product chemical diversity and begin to formulate hypotheses about their biogenesis. Metabolomics data from *Prunella vulgaris,* a species that contains a wide range ofantioxidant, antiviral, tumoricidal and anti-inflammatory constituents, provide a case study of obtaining biosystematic and developmental fingerprint information from metabolite accumulation data in a little studied species. *Digitalis purpurea,* well known as a source of cardiac glycosides, is used to illustrate how integrating metabolomics and transcriptomics data can lead to identification of candidate genes encoding biosynthetic enzymes in the cardiac glycoside pathway. Medicinal Plant Metabolomics Resource (MPM) [1] provides a framework for generating experimentally testable hypotheses about the metabolic networks that lead to the generation of specialized compounds, identifying genes that control their biosynthesis and establishing a basis for modeling metabolism in less studied species. The database is publicly available and can be used by researchers in medicine and plant biology.

## 1. Introduction

Humans have relied for millennia on natural products for relief of pain and improvement of health. Specialized metabolites from plants serve as rich resources for drug development. The molecular and physiological effects of medicinal plant extracts and components are often characterized in research studies of mammalian systems; almost 100 plant-derived compounds were in clinical trials in 2007, and as of 2008, 68% of all pharmaceuticals were plant derived or plant inspired [2]. 

In part because they lack mobility, plants have evolved chemically-based strategies for defense and attraction [3,4]. As a consequence, even low levels of tens of thousands of the hundreds of thousands of metabolites that are synthesized across the plant kingdom interact with mammalian signaling networks via variety of molecular mechanisms. However, metabolic diversity is poorly characterized for most species that are used medicinally and indeed for plants in general. In addition, understanding of the molecules and metabolic pathways that lead to the formation of already-known plant-derived medicinal compounds is still incomplete. Modeling of metabolism requires computational technologies acting on multidimensional data, integrated with informed biological understanding of metabolites and pathways. In the case of medicinal plants (*i.e.,* non-model-species), such data are scarce and difficult to integrate into a meaningful biological framework. One feature that can facilitate studies of plant metabolites and the corresponding pathways is that the content and profile of metabolite accumulation vary widely with developmental stage, cell and tissue type, genotype, and environmental perturbation [5,6,7]. A metabolomics-based analysis of natural products across multiple conditions is a first step towards elucidating the associated metabolic pathways and identifying enzymatic and regulatory genes associated with these pathways. 

The development of publicly-available genomic, transcriptomic, and more recently, metabolomic, flux and proteomic data sets for model organisms has accelerated the understanding of metabolism and metabolic networks [2,8,9,10,11,12,13,14]. Analogous data sets for medicinal plants will similarly revolutionize how researchers approach, decipher, and model the accumulation of medicinal compounds, and consequently enable the more effective development and utilization of medicinally active plant metabolites. This manuscript describes an information-rich database platform for medicinal plants (Medicinal Plant Metabolomics Resource (MPMR, [1]) assembled through a large-scale, collaborative effort, and illustrates how such an investment can impact many who work in the fields of medicinal plant chemistry, biochemistry, metabolic modeling, and drug development. This resource is linked to transcriptomics data for the same samples (Medicinal Plant Metabolomics Resource (MPGR; [15]). The overall effort is part of the Medicinal Plant Consortium (MPC), an NIH-supported project including 13 collaborating research units from 7 institutions focused on providing transcriptomic [15] and metabolomic [1] resources for 14 key medicinal plants to the worldwide research community for the advancement of drug production and development.

MPMR is meaningful to the wider research community because it is available to all researchers for evaluation. A major challenge in evaluating complex datasets is how to best visualize these data to readily extract new knowledge. Here, we detail the public database MPMR, and we illustrate with test cases as to how the MPMR database can be used to extract information and provide a framework for researchers to generate experimentally testable hypotheses about the metabolites and metabolic networks that lead to the generation of specialized compounds. 

## 2. Results and Discussion

Metabolomics data represent deep and comprehensive measures of the levels of metabolites in a defined tissue. In order for metabolomics data to be seamlessly integrated with other global molecular datasets that define the biological status of tissue(s), it needs to be organized and normalized in a standard format that enables cross-referencing with multiple datasets. Integral to this organization are the associated metadata that define the biological status of the tissue under analysis, and the methods used to extract and analyze the metabolites. The MPMR database and interface provide the ability to organize metabolomics data and metadata. The user interface and tools for MPMR were shaped in part by discussions among various participants of the MPC. 

Researchers can investigate the data using the tools within MPMR, or download it for additional statistical or bioinformatics analysis. These data can inform researchers who are planning detailed biochemical studies or who are devising a framework for a metabolic model for a medicinal plant species. 

An analytical approach to metabolomics used for many species in MPMR is Liquid Chromatography-Time-Of-Flight Mass Spectrometry (LC/TOF-MS); this method employs an information-rich strategy, termed multiplexed collision-induced dissociation (multiplexed CID) [16,17] that acquires mass spectra from 4 (or 5) different collision energies on the time-frame of ultrahigh performance liquid chromatography (UHPLC). LC/TOF-MS generates accurate molecular and fragment masses for all detected compounds, including low abundance intermediates, and yielded lists of several hundred to several thousand detected signals for each sample [18]. In deep metabolite profiling of plant material, the number of detected metabolites far outstrips the number of known natural products. 

**Figure 1:**
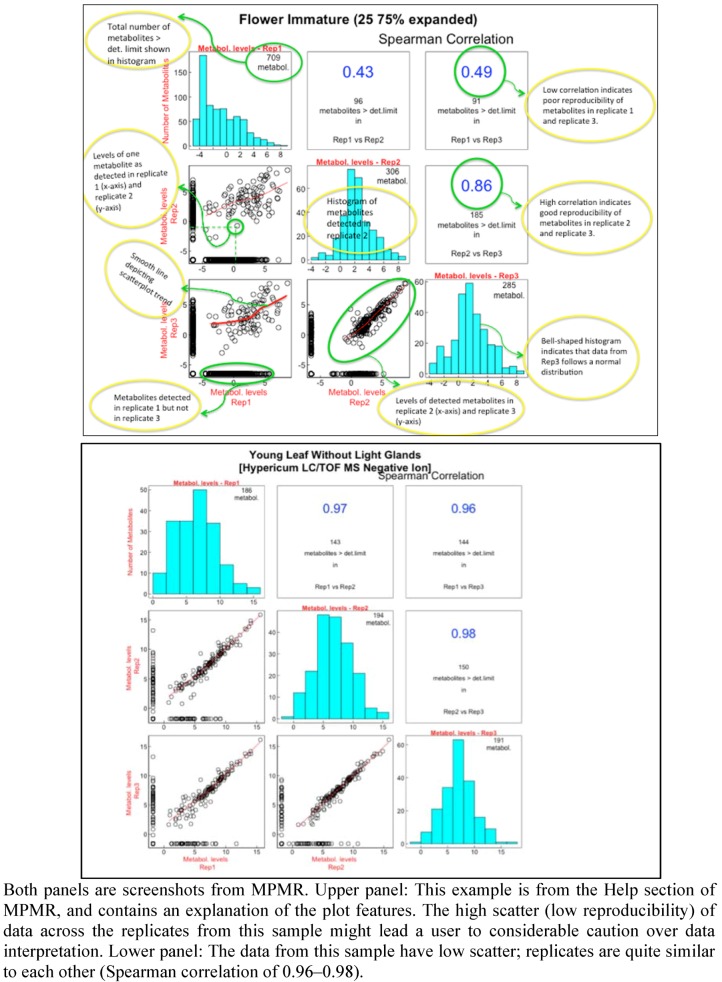
Scatterplots providing a rapid method for assessing the quality of metabolite determinations.

In addition, a variety of Gas Chromatography-Mass Spectrometry (GC-MS) protocols were used for metabolite analysis. These incorporate a more targeted approach designed to highlight classes of known metabolites. The GC-MS platforms may detect hundreds of metabolites, many of which are also not identified.

Metabolomic analysis of medicinal plant samples yields a rich resource of information, and one that is often ripe with surprises. To enable these data to be used by the community, the data are exported to and accessible through MPMR. Metadata describing the plant material, extraction, separation and analytical techniques are added. Various features are incorporated into MPMR to facilitate data exploration. An interactive comparison of the replicates of each organ analyzed, using scatterplots representing pairwise comparisons of replicates combined with Spearman correlations and bar graphs, enables the user to quickly assess the general quality of the data (Figure 1). MPMR can be searched by key words and molecular masses, and the data can be sorted in a variety of ways. Linked plots and tables enable the user to track the data from various vantage points. Three case studies of how MPMR can be used to inform a researcher are presented.

### 2.1. Case Study: Deep Metabolic Profiling of Camptotheca acuminata as an Approach for Development of Hypotheses about the Camptothecin Biosynthesis Network

**Figure 2:**
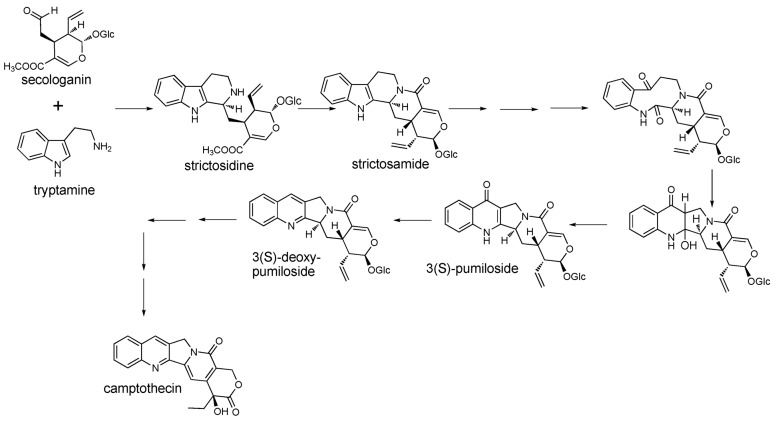
Proposed steps in the intermediate and late stages of camptothecin biosynthesis.

LC/TOF-MS analyses of *C. acuminata* Decne. (Cornaceae) (Chinese happy tree, source of the anticancer compound camptothecin) revealed more than 50 abundant peaks, with more than 1900 additional signals extracted into the metabolite database. Camptothecin is a quinoline alkaloid derived from an indole terpene alkaloid biosynthetic pathway [19]. The early steps in camptothecin biosynthesis involve formation of terpenoid precursors (through 10-hydroxygeraniol) and tryptamine, and these pathways must converge to form more complex intermediates (Figure 2). However, evidence for intermediate steps in the pathway has remained elusive, with several putative intermediates missing from the most extensive literature report of *C.* *acuminata* metabolite profiles [20]. 

**Figure 3:**
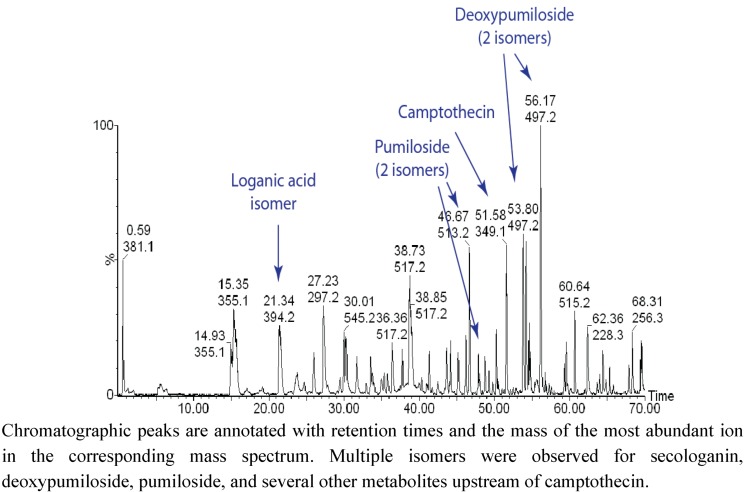
UHPLC/TOF-MS profile of metabolites extracted from *C. acuminata* bark.

The complexity of the *C.* *acuminata* metabolome is reflected in Figure 3, which displays an LC/TOF-MS chromatogram of a bark extract. One of the more interesting realizations derived from these data is that several key intermediates were present as mixtures of isomers, and in many cases, abundances of fragment ions were not sufficient to distinguish these isomers. In the latter stages of the pathway, pairs of isomers were detected for strictosamide, pumiloside, deoxypumiloside, and other metabolites including a putative ketolactam. The isomeric metabolites have yet to be purified for complete structure elucidation, but one possibility is that they are stereoisomers with different configurations at the 3-position. The similarity in the ratios of isomers indicates that enzymes that catalyze these transformations may not exhibit much stereoselectivity in substrates.

**Figure 4:**
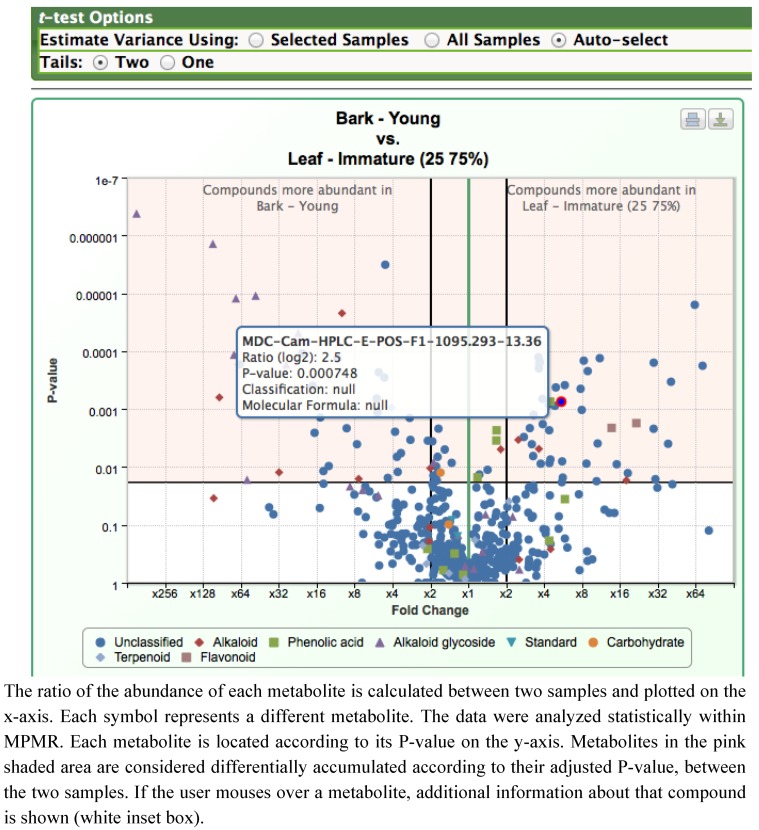
Volcano plots illustrating the difference in metabolite levels between bark and partially expanded young leaves, as shown in a screenshot from MPMR.

A rapid way to compare the difference between two datasets is a volcano plot, and we have found this approach to be very useful for visualizing metabolomics datasets and quickly identifying metabolites that are significantly altered between the two datasets (Figure 4). In these plots, the ratio of the abundance of each analyzed metabolite is calculated between two samples; this ratio is plotted on the x-axis. The metabolomics data are analyzed statistically using a t-test, and each metabolite is placed according to its P-value on the y-axis. Figure 4 represents metabolite abundances in young bark *versus* immature leaves (25-75% expanded). This plot indicates that a subset of alkaloids, and all of the detected alkaloid glycosides, are more abundant in young bark; however, several isomers of camptothecin are more predominant in young leaves. The plot also provides an indication of the statistical significance of the difference in abundance for each metabolite.

**Figure 5:**
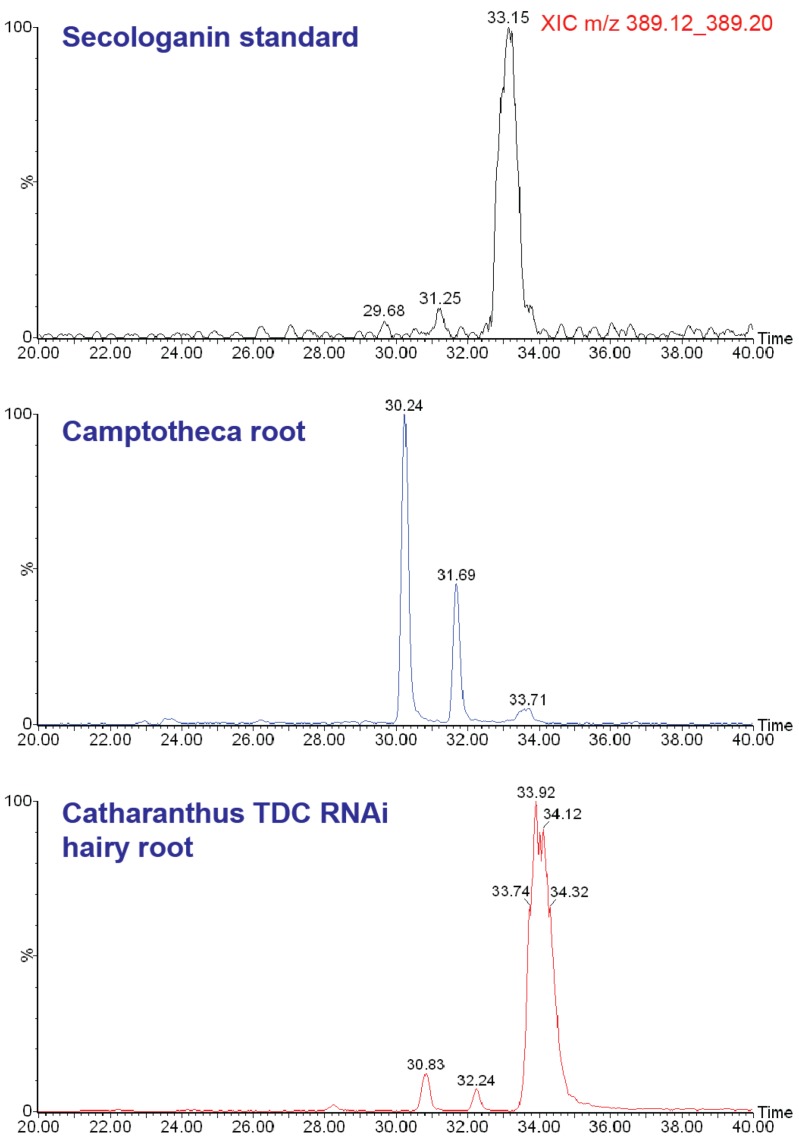
Extracted ion LC/MS chromatograms of [M+H]^+^ for (top) authentic secologanin standard, (middle) *C. acuminata* root extract, and (bottom) *C. roseus* RNA interference line of hairy root culture. The third peak in the latter two corresponds with secologanin, whereas the two earlier eluting peaks (in both *C. acuminata* and *C. roseus*) are isomers indistinguishable from molecular or fragment masses.

Additional review of the *Camptotheca* metabolome reveals several more surprises. First, the proposed terpenoid intermediate secologanin [21,22] is barely detectable in any of the *C. acuminata* organs and tissues that we analyzed. This led us to conduct a follow-up analysis of several plant extracts, by using a slower UHPLC solvent gradient and a longer chromatographic column to better resolve isomeric metabolites (Figure 5). Extracted ion chromatograms for the [M+H]^+^ ion of secologanin contain two peaks corresponding to secologanin isomers with retention times that are distinct from an authentic secologanin standard and from the major secologanin isomer in an extract of *Catharanthus roseus*. 

### 2.2. Case Study 2: Using Metabolite Levels across Accessions and Organs of Prunella vulgaris to Investigate Intraspecific Diversity

Commonly known as “selfheal” or “heal-all,” *P. vulgaris* L. (Lamiaceae) is a low-growing perennial herb native to a large part of the Northern Hemisphere. It is a relatively poorly characterized species that has recently been shown to have a wide variety of bioactivities. Its dried inflorescences have a long history of use in traditional Asian and European medicine [23,24,25,26] as a remedy for cancer, sore throat, fever, and wounds. Recent data indicate that this species has potential to become an economically important medicinal herb through the wide scope of biological activities associated with *P. vulgaris* extracts [27,28,29,30,31,32,33,34]. Diverse bioactive compounds have been characterized from these extracts. Aqueous extracts display antioxidant, antiviral, tumoricidal and anti-inflammatory properties and are known to contain polyphenols and complex carbohydrates. *P. vulgaris* polysaccharides have exhibited antiviral, immunomodulatory and anti-tumorigenic activity [28,34,35,36,37,38,39], whereas phenolic constituents, such as rosmarinic acid, have antioxidant as well as immunomodulatory activity [33,40,41,42]. Ethanolic extracts contain triterpenes and flavonoids [33,43,44], and several such compounds and extracts have significant anti-inflammatory activity [45,46]. 

Because *P. vulgaris* has recently been shown to have a wide variety of bioactivities, but little research characterizes biochemical aspects of this species, the goal of this study was to provide information on metabolites across different accessions and developmental stages. These data would provide a potential basis for standardization for this species, and would enable a researcher to gain a quick understanding of what material they might want to employ in bioactivity assays or use to investigate the metabolic reactions and networks that lead to the specialized components. We also use the data to compare each accession by its metabolomic “fingerprint” and provide further understanding of their provenances.

**Figure 6:**
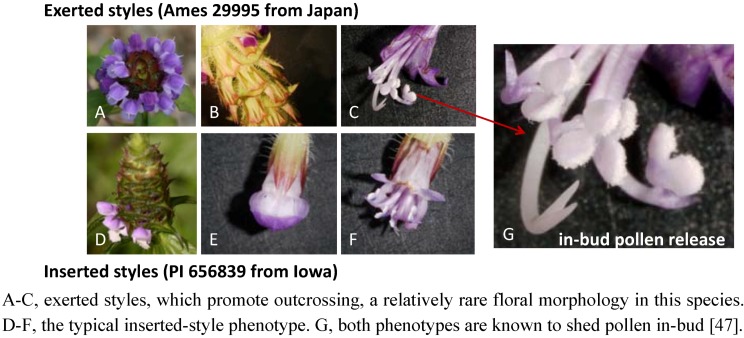
Variation in the breeding system of *Prunella vulgaris* can be visualized by this comparison of accessions Ames 29995 and PI 656839.

Typically, *P. vulgaris* populations display patterns of floral morphology that reflect a tendency towards self-pollination, but variants that promote outcrossing have also been noted (Figure 6) [47]. A mixed, but primarily autogamous, breeding system can result in the evolution of well-differentiated, local populations [48] that vary in adaptation, as noted in *Prunella* by Bocher [49], Nelson [50], Schmid [51] and Winn and Gross [52], and this could include adaptations in metabolite composition. Brindley *et al.* [53] demonstrated that there is significant variation in antiviral properties among different *P. vulgaris* accessions cultivated under a common set of field conditions. In a separate study, the bioactive compound, rosmarinic acid, was evaluated in shoots of eleven *P. vulgaris* accessions; these shoots were shown to differ by over 10-fold in concentrations of rosmarinic acid (Berhow *et al.*, personal communication). These results indicate that there is likely a substantial genetic variation among *P. vulgaris* populations, at least for metabolites that would be likely to confer differences in bioactivity and pharmacological efficacy. Season of harvest can also influence metabolite composition, as evidenced by Chen *et al.* [54], who documented seasonal changes in rosmarinic, ursolic, and oleanolic acid concentrations in dried *Prunella* inflorescences. 

With the increasing emergence of information about the properties of this species in the last decade, *P. vulgaris* was incorporated into the medicinal plant germplasm collection conserved by the USDA-ARS North Central Regional Plant Introduction Station (NCRPIS, Ames, IA) and from 2007 to 2011 was one of three medicinal-plant genera being studied in Iowa by the Center for Research on Botanical Dietary Supplements [55]. Currently, the NCRPIS conserves 48 *Prunella* accessions from both Old and New World origins (USDA-ARS GRIN [56]). 

**Figure 7:**
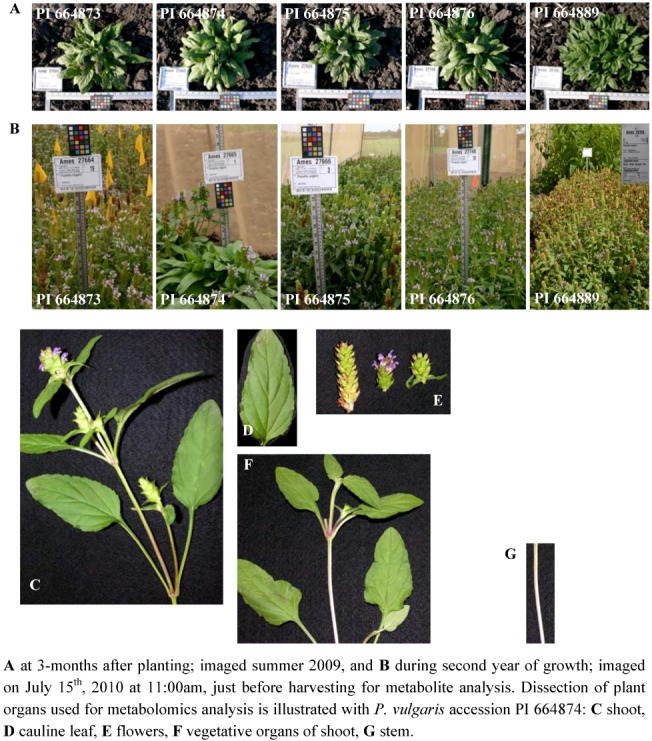
*Prunella vulgaris* accessions growing at the Plant Introduction Station, Ames, IA.

Five *Prunella vulgaris* accessions were chosen for metabolite fingerprinting analysis, based on diverse locations from which they were sourced (Table 1). Four of these accessions were originally sourced as wild populations from four different locations in North America, and one was collected from a site in Eastern Europe (South Ossetia, Georgia). Seeds collected from these populations were germinated and planted at the USDA North Central Regional Plant Introduction Station, Ames, IA. Figure 7 shows the appearance of these plants during the first two years of growth. By approximately 3-months after planting, the Georgia accession (PI 664889) showed a clearly distinguishable morphological difference from the North American accessions; the former plants presenting a denser appearance. The majority of the plants did not flower during the first year of growth. However, in the second growth season, these plants flowered, and we collected intact aerial organs as illustrated in Figure 7. The organs that were subjected to metabolomics analyses were: shoots, cauline leaves, flowers, vegetative organs of shoots, and stems; metadata on these samples are provided at the MPMR database. 

**Table 1:** List of *Prunella vulgaris* accessions used in this study, their geographical origin and date of collection. All accessions were grown at the USDA Plant Introduction Station, Ames, IA.

ACNO	Place of collection	Records
PI 664873 (Ames 27664)	North Carolina, United States	11/19/2004
PI 664874 (Ames 27665)	North Carolina, United States	11/19/2004
PI 664875 (Ames 27666)	North Carolina, United States	11/19/2004
PI 664876 (Ames 27748)	Missouri, United States	12/29/2004
PI 664889 (Ames 29156)	South Ossetia, Georgia	06/16/2008

#### 2.2.1. Platforms Used in the Detection of *Prunella* Metabolites

Fingerprint analysis of the metabolomes of the five *Prunella* accessions used a combination of metabolic profiling strategies: a non-targeted metabolomics analysis and three targeted metabolite-profiling platforms. For non-targeted analysis, we used GC-MS based analysis of metabolite extracts. The advantage of this method is that it is highly sensitive, relatively easy to apply, and due to its history of use, chemical identification of detected compounds is facilitated by predictive rules of fragmentation during spectroscopy. This has led to the development of rigorous mass-spectral libraries, which facilitate chemical identification [57,58]. The limitation of this method is that only small compounds, of less than about 1000 Da, can be analyzed, and chemical derivatization is needed to facilitate the volatilization of compounds into the gas-phase. 

The three targeted metabolite-profiling platforms that had previously been used in analyzing the metabolomes of *Arabidopsis* were used in these analyses, and they revealed the relative abundance of 21 amino acids, 119 surface lipids, and 83 fatty acids, and the non-targeted metabolomics platform detected 222 metabolites (metadata via the extraction protocols and the identification of these metabolites are provided in the MPMR database). This approach of combining different analytical platforms enabled us to evaluate the relative abundance of nearly 450 *Prunella* metabolites. The rationale for combining non-targeted and targeted metabolite analysis is complex, and is aimed at maximizing the researcher’s ability to analytically access the “entire” metabolome of the samples, while also accurately annotating the chemical identities of many detected metabolites. We have chemically identified about 1/3 of the 450 *Prunella* metabolites that were detected, and most of these were detected in the targeted metabolite analysis platforms. Although network topologies can be best determined once chemical identities of metabolites are ascertained, all data can be used as a fingerprint to evaluate differences and similarities among the samples. 

#### 2.2.2. Data Visualization and Evaluation of *Prunella vulgaris*

**Figure 8:**
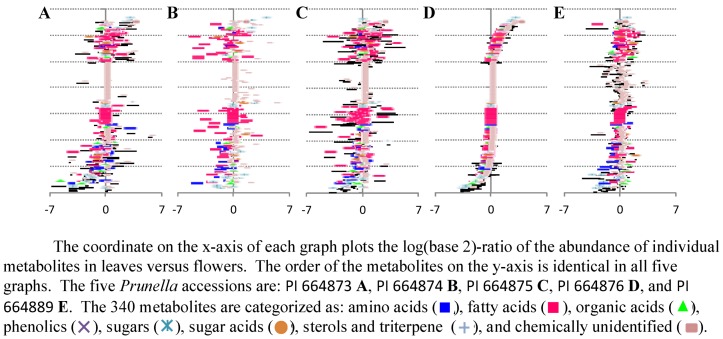
Differential accumulation of 340 metabolites between leaves and flowers among five different *Prunella* accessions.

The goal for this case study was to visualize the metabolites (out of the 450 that were evaluated) that accumulate at different levels among the different accessions and organs, and to use these data to evaluate the biological basis for these divergences. A ratio plot (Figure 8) is a standard method that enables an experimenter to focus on those metabolites that are most altered in abundance between two samples. Examples of such graphs are shown in Figure 8, which plots the ratio of metabolite abundances between cauline leaves and flowers in the five *Prunella* accessions. Additional insights into the chemical nature of the metabolites are provided by the color and shape of the symbol that is used to represent each metabolite in the graph, and the interactivity of the graph with the database. The experimenter can at a glance identify those metabolites that are most abundant in leaves (the ones that plot most distally from the x-axis origin in the positive direction), and those metabolites that are most abundant in flowers (the metabolites that plot most distally from the x-axis origin in the negative direction). This provides a quick look at the profile of each metabolite across the organs and accessions. 

However, while ratio plots are useful because of their simplicity, each one can only compare two samples. To analyze data using statistics specific for that dataset, we compare similar plots from all pairwise sample-set comparisons, and combine the resultant graphs. This provides a means of visualizing the data in an orthogonal manner. In all the graphs shown in Figure 8, the order of the metabolites on the y-axis is identical, and therefore one can directly compare all five graphs, and thus visualize the effect of genetic variation on the development of metabolic differences between leaves and flowers. A comparison of Figure 8D and Figure 8E is an example. Because the order of the metabolites on the y-axis is the same, these two graphs are directly comparable. The two panels plot plots of the log-ratio abundance of metabolites in leaves *versus* flowers in *Prunella* accessions PI 664873 (8D) and PI 664889 (8E). It is immediately obvious that metabolites accumulate differentially in terms of their developmental profile (leaves *versus* flowers), but this developmental profile is likely affected by the genetic diversity that is inherent in the two different accessions that are being compared. 

**Figure 9:**
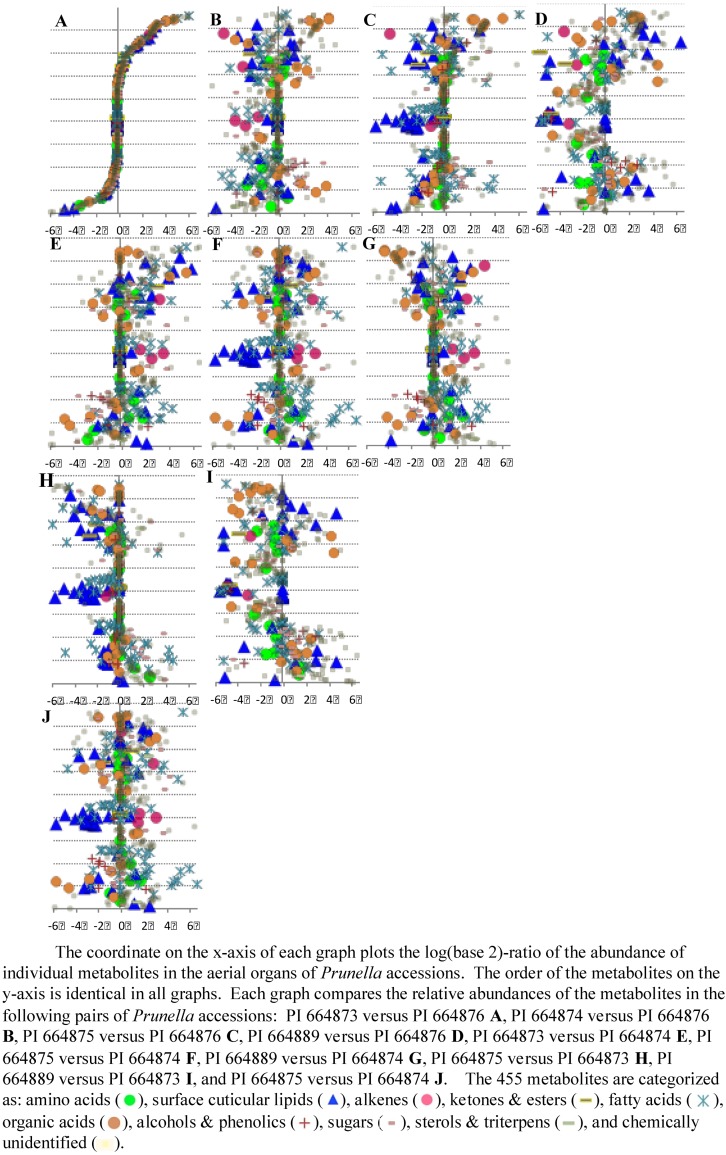
Differential accumulation of 455 metabolites between different pairs of *Prunella* accessions.

Figure 9 illustrates an approach to visually compare accessions based on relative metabolite abundances, and use these data as fingerprints to distinguish the metabolic differences among the five *Prunella* accessions. The ten graphs in this figure plot the relative abundance of all the detected metabolites in all organs assayed by using the abundance of the metabolites in each accession as the denominator in the calculation of the log-ratio values. In these graphs, the order of the metabolites is identical, and therefore the pattern of the graphs is directly comparable. Therefore, the metabolic relationship among the five accessions becomes a problem of pattern recognition – namely which two patterns are most similar or most different from the other. We used a combination of pattern-recognition algorithms and statistical evaluations to address this question.

#### 2.2.3. Statistical analysis of the metabolomics data to evaluate systematic relationships among *Prunella* accessions.

An approach to evaluate the metabolic profiling data is to integrate the relative abundance of all or a subset of the evaluated analytes, and calculate statistical distances among the biological samples (i.e., the five *Prunella* accessions). Although there are many choices of statistical distance measures, weighted Manhattan distance measure (D_ij_) is convenient, and has proven useful in analyzing metabolomics data [59,60,61]. The weighted Manhattan distance measure computes the dissimilarity between a pair of samples via the equation:

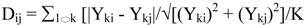

where: D_ij_ is the weighted Manhattan distance between sample I and J; Y_ki_ is the abundance of metabolite k in sample I, and Y_kj _is abundance of metabolite k in sample J; K is the total number of metabolites. The term √[(Y_ki_)^2^ + (Y_kj_)^2^] estimates the standard deviation of the difference in abundance.

One property of this distance measure is its invariance to multiplicative rescaling of metabolite abundance data, which means the contribution of any metabolite to the value of D_ij_ is the same no matter what the datum point determines (i.e., a peak area, a relative abundance, or an absolute concentration), as long as each of these quantities can be converted into another by multiplying by a constant factor. Moreover, each metabolite data point contributes equally to the calculation of the value of D_ij_ irrespective of the magnitude of its abundance value. The statistical distance (D_ij_) between each pair of distinct samples ranges between zero and 1.0. When the two samples express the identical abundance for each metabolite the D_ij_ = 0, and when the two samples have completely different constituents, D_ij_ = 1.0. 

**Figure 10:**
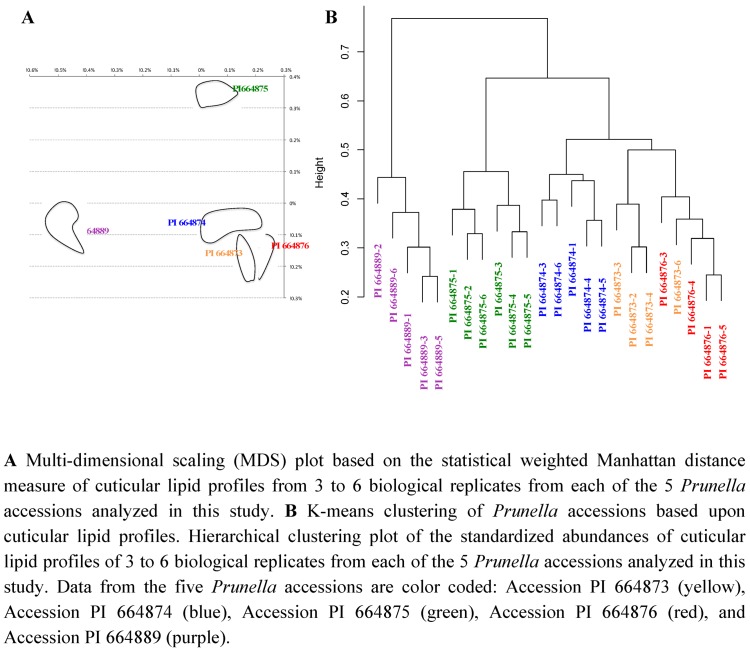
Statistical distances among *Prunella* accessions based upon cuticular lipid profiles from vegetative organs of shoots.

Based upon these distance calculations, relative statistical distances among the five *Prunella* accessions can be visualized by using multi-dimensional scaling (MDS) plots. The MDS plot of Figure 10A represents 25 samples, composed of five experimental datasets generated from five *Prunella* accessions. In this plot, the distance between a pair of points represents the weighted statistical distance between a pair of *Prunella* samples based upon the profiling of the vegetative organs of shoots for cuticular lipids. The distances between points in the MDS plot are the best two-dimensional approximation to all pairs of D_ij_ values in the distance matrix. This representation indicates that biological replicates with each accession cluster proximal to each other, and the underlying metabolic differences among these accessions can be used to distinguish each accession. Namely, of the 4 North American accessions, PI 664876, PI 664873 and PI 664874 resemble each other more closely than they do PI 664875, and these two groupings are equally distinct from the Georgian accession (PI 664889). Although the latter would be expected based upon geographic provenance, and parallels the morphological differences among these accessions, the difference between PI 664875 and the other accessions is somewhat unexpected, and would not have been revealed without metabolic profiling. 

A similar conclusion about the relationships among the *Prunella* accessions is reached by a different statistical analysis of the cuticular lipid data, K-means clustering [62] of the standardized abundances. In this analysis, each abundance level was normalized by dividing each value with the maximum abundance of that metabolite in each sample. Hierarchical clustering of the standardized abundances identified three accession groups (Figure 10B). The most distinct cluster contains the biological replicates from the Georgian accession (PI 664889), and the samples from the North American accessions PI 664876, PI 664873 and PI 664874 cluster relatively closely together, whereas accession PI 664875 is placed equidistant from the other two groups, a conclusion much like that reached from the MDS plot. 

### 2.3. Case Study: Combined Metabolomics and Transcriptomics of Digitalis purpurea for Hypothesis Development

**Figure 11:**
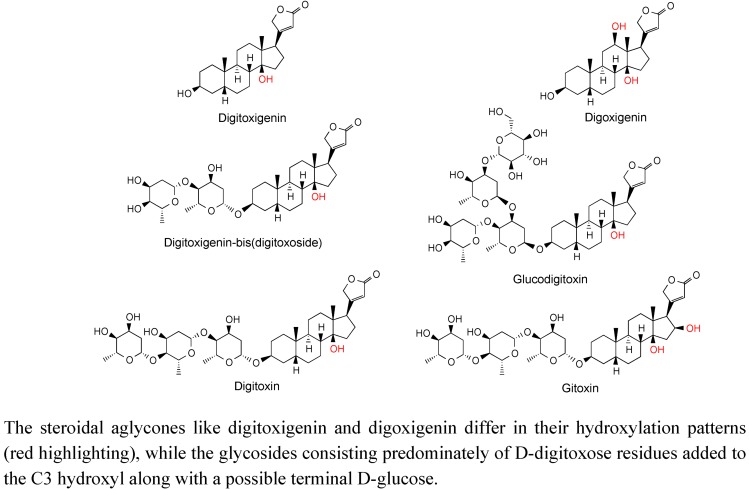
Structure of cardenolides isolated from diverse *Digitalis* species.

Cardenolides are a large and structurally diverse class of steroid derivatives found in select plant families, such as the Apocynaceae, but probably are more readily recognized for their association with foxglove (*Digitalis* species, Plantaginaceae) [63], and their therapeutic value as cardiac muscle stimulants [64]. For instance, *Digitalis purpurea* L. and *D. lanata* Ehrh. are credited as being some of the oldest herbal remedies for specific cardiac ailments, with a record of use dating from 1785 [65]. The biologically active chemicals found in *Digitalis* species were identified more than 50 years ago [66] and consist predominately of mono-, di- and tri-glycosides of specific steroid skeletons, such as digitoxigenin and digoxigenin (Figure 11). These compounds are still obtained by extraction from foxglove.

Cardenolides belong to the larger chemical family of cardiac glycosides, which also include bufadienolides. Cardenolides and bufadienolides are both extremely toxic substances that possess multiple biological activities [64], but both are known to bind at nM concentrations to Na^+^/K^+^-ATPases, sodium export transporters associated with maintenance of the ionic balance of ions across cell membranes [63,64] . Inhibition of these transporters strengthens the contraction of cardiac muscle cells at low, therapeutic concentrations, but is toxic and often lethal to many of the herbivores and other chewing pests [63] that attempt to consume the plants and animals producing cardiac glycosides [67]. The major difference between these two classes of chemicals is that cardenolides contain a five-membered lactone ring while the bufadienolides contain a six-membered lactone ring [64]. Equally intriguing, cardenolides are found largely in plants, while the bufadienolides are associated with skin glands of toxic toads [63]. As argued by Dobler *et al.* [63], given the structural similarities between the cardenolides and bufadienolides, the development of the biosynthetic machinery for cardenolides in plants and bufadienolides in amphibians may represent an example of convergent evolution.

The pharmaceutical availability of cardenolides has depended upon their natural sources because of difficulties associated with their chemical syntheses [68]. Even more surprising is that the biosynthetic pathways for these diverse cardenolides have not been completely worked out, and there are numerous outstanding questions about their biosynthesis and accumulation. A better understanding of the biosynthetic pathway(s) per se should be useful in modeling cardenolide accumulation, in the design of molecular genetic screens to improve cardenolide end-product yield, and to develop plant lines accumulating a greater diversity of cardenolide products that could be screened for new or enhanced biological activities. 

**Figure 12:**
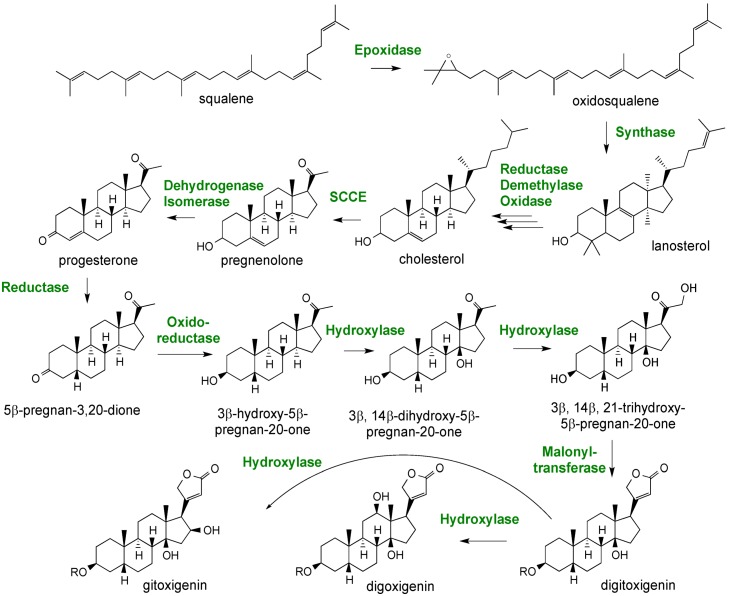
A proposed pathway for digitoxin biosynthesis in *Digitalis* spp.

Although experiments feeding intermediates have demonstrated that some of the proposed reactions do occur *in planta* and a few of the enzymes have actually been measured in *in vitro* assays [69], many of the steps depicted remain to be experimentally confirmed. Lanosterol is depicted as the first committed intermediate to cardenolide biosynthesis. Oxidosqualene is more commonly channeled to cycloartenol, the well-established precursor to stigmasterol, sitosterol and campesterol, the dominant sterols found in all plants, rather than to lanosterol. However, only lanosterol and not cycloartenol has been demonstrated to be converted to cholesterol, and there is clear experimental precedent for the conversion of cholesterol to the pregnane family of steroids in mammals [64]. Hence, most depictions of the digitoxin biosynthetic pathway propose the lanosterol to cholesterol steps as intermediates, yet there is scant experimental evidence for this. Furthermore, only a couple of the genes encoding for enzymes in later steps of the pathway have been functionally characterized, such as that for the progesterone 5β-reductase [64].

A putative, but partial pathway for cardenolide biosynthesis is shown in Figure 12; it is based largely on earlier precursor feeding studies and inferences taken from similar biochemical transformations occurring in mammalian steroidal biosynthetic pathways [69]. The proposed pathway also provides a means for directing attention to questions remaining to be resolved. For instance, are the cardenolides derived from cholesterol or perhaps are they derived from other phytosterol precursors, such as stigmasterol or sistosterol? While it has long been known that digitoxins accumulate in leaves, it is not clear if the site of synthesis differs from the site of accumulation. Are intermediates or end-products translocated between tissues? Once a biochemical pathway for digitoxins has been worked out, another question is how might cardenolide biosynthesis be regulated spatially and temporally relative to those pathways directing the sterol biosynthesis important for membrane biogenesis? Are there separate pathways localized to distinct intracellular compartments, or is there a central pipeline with bifurcation points where intermediates might be diverted to the biosynthesis of one class of sterols *versus* another? 

To address some of these questions, we developed metabolomics and transcriptomics resources for *Digitalis* with the long-range aim of applying these tools to define and validate a cardenolide biosynthetic pathway. Metabolite profiles assessed by high resolution LC/TOF-MS yielding approximately 2000 different metabolites were recorded for each plant extract, with metabolite validation from triplicate biological replications and duplicate technical replicates. 

**Figure 13:**
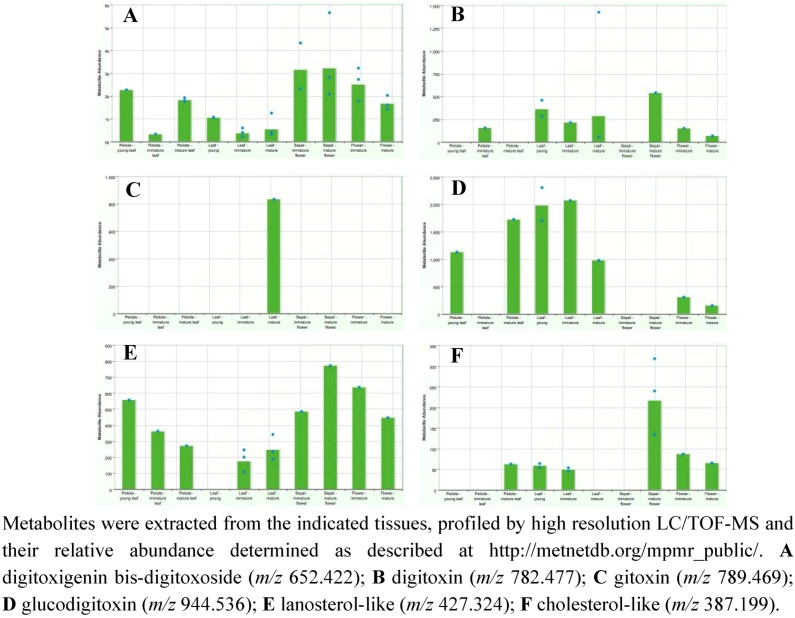
Levels of select cardenolides and putative biosynthetic intermediates in various tissues of *Digitalis purpurea*.

Figure 13 provides screen shots from MPMR of the metabolic profile for select metabolites in various tissues of *D. purpurea* as a means for determining where cardenolides and putative intermediates might be accumulating. Four of the metabolites known for their pharmacological activities [63] were specifically monitored: digitoxigenin bis-digitoxoside; digitoxin; gitoxin; and glucodigitoxin. Interestingly, while all 4 of these metabolites have been found in leaf extracts [66], their distribution across diverse stages and organ types shows some striking differences. For instance, while digitoxigenin bis-digitoxoside levels in leaves are significant, much higher levels are observed in sepals and flowers (Figure 13A). In contrast, glucodigitoxin, another potent pharmacological compound, appears to accumulate preferentially in leaves with virtually none in sepals and lesser amounts in flowers (Figure 13B). Such an observation suggests that the glycotransferases specific for elaborating the digitoxigenin bis-digitoxoside to glucodigitoxin are present and active in leaves, but absent in sepals and only modestly in petals.

The *D. purpurea* metabolome was also screened for lanosterol-like and cholesterol-like metabolites likely to be intermediates in the pathway, with the idea that if the digitoxigenin steroid skeleton were synthesized in only one specific plant part, and then distributed throughout the plant for tissue-or organ-specific elaboration, one might expect these metabolites to accumulate exclusively in the region where their synthesis occurred. From the metabolite profiles (e.g., Figure 13E and F), this possibility seems unlikely. Instead, the lanosterol-like metabolites appear to be present almost exclusively (Figure 13E) or, for the cholesterol-like metabolite, predominately (Figure 13F) in those plant parts where the cardenolides accumulate. These results would argue that the enzymes for lanosterol biosynthesis and the conversion of lanosterol to cholesterol should be present in all these tissues, and most abundant or active in leaf and floral tissues. 

**Figure 14:**
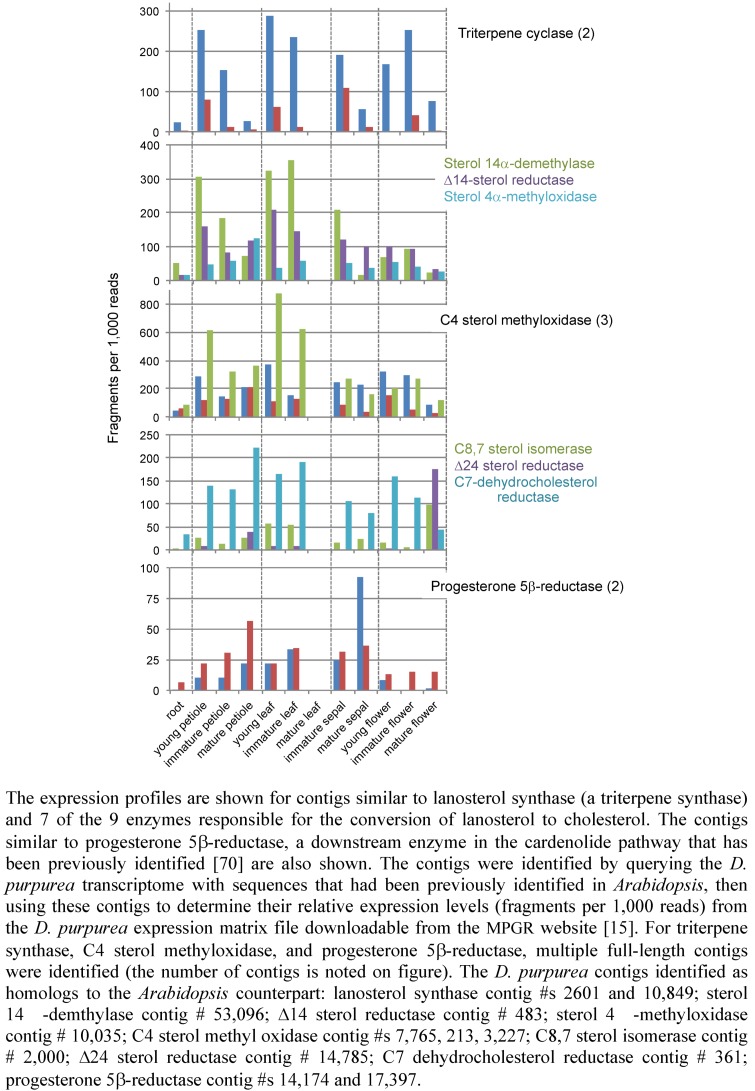
The expression profile of contigs from *D. purpurea* with sequence similarity to cardenolide metabolic enzymes.

We screened the *D. purpurea* transcriptome for candidates for genes encoding the enzymes responsible for the biosynthesis of lanosterol and its conversion to cholesterol, and then examined the relative abundance of each transcript in various organ types, as determined by a count of the number of respective sequence reads found for each assembled contig [15] (Figure 14). The progesterone 5β-reductase contigs served as a technical control in this analysis, because only this gene and one other relevant biosynthetic gene have been reported in the literature [70]. As illustrated in Figure 14, two independent contigs for the progesterone 5β-reductase were identified, each of which differ somewhat from the progesterone 5β-reductase gene previously characterized by Herl *et al.* [70]. The contigs identified in the *D. purpurea* transcriptome are more than 72% identical to the previously characterized gene, with their similarity exceeding 83%. The two contigs exhibit greater than 78% identity to one another. 

The apparent abundance of these two transcripts was contrary to our expectations from the metabolite-profiling analysis. Based on the relatively high level of end-product cardenolides in leaves and floral organs, we anticipated that the level of the progesterone 5β--reductase transcript would mirror these metabolite levels. Instead, the reductase transcript level is constitutive across all the organs examined, which would suggest that some other step(s) in the pathway are rate-limiting for end-product cardenolide accumulation. 

The other question we posed of the *D. purpurea* transcriptome data is whether the expression level of genes for lanosterol and cholesterol biosynthesis are correlated with cardenolide accumulation. Essentially, we are asking if the steroidal skeleton of the cardenolides could be derived from a cholesterol precursor, consistent with the chemical rationalization for cardenolide biosynthesis (Figure 12). 

Two full-length triterpene synthase contigs were found when we queried the *D. purpurea* transcriptome with a lanosterol synthase gene from *Arabidopsis* [71]. The predicted proteins encoded from these contigs were 54 and 65% identical to the *Arabidopsis* lanosterol synthase, with similarity scores exceeding 70%. Interestingly, one of the contigs exhibited a relatively high level of expression in all tissue types, while the second contig demonstrated a lesser and variable abundance pattern. For instance, this second contig was more abundant in young or immature petioles, leaves, sepals and flowers than in the mature forms of these tissues. This sort of pattern might be consistent with a role for the enzyme encoded by this gene in cardenolide biosynthesis because metabolite accumulation commences in these immature tissues and continues on into the later stages of development. 

The conversion of lanosterol to cholesterol is a 19-step process requiring nine different enzymes, many of which participate in multiple steps and several that participate in analogous reactions at different points in the pathway [72]. When the *D. purpurea* transcriptome was queried for gene homologs coding for these enzymes, contigs for 7 of these genes were identified with multiple contigs found for the C4 sterol methyl oxidase (Figure 14). Contigs were not found for the C3 sterol dehydrogenase, nor for the C3 keto steroid reductase. Our survey of the *D. purpurea* transcriptome is by no means comprehensive or complete at this stage, because our screen was limited by the query sequences available for use. For instance, the transcriptome was screened for C3 keto steroid reductase contigs based on yeast and rat steroid reductase sequences, but no homologs were detected. This does not mean the C3 keto steroid reductase is absent from *D. purpurea*. It could be that such a gene arose independently with little or no sequence relationship to the yeast or rat genes, or that the genes have diverged widely in sequence.

The transcript abundance profiles for these *D. purpurea* contigs were quite variable (Figure 14), and it is difficult to discern the extent to which any of the transcript profiles exhibited a pattern correlating with the accumulation patterns for the lanosterol or cholesterol-like metabolites (Figure 13). If anything, the transcript profiles would suggest that *D. purpurea* is likely to have the enzymological capacity for the conversion of lanosterol to cholesterol in many organs; ascertaining whether any of these enzymes are limiting or regulatory for cardenolide biosynthesis must await more detailed studies including measurement of the respective enzyme activities in each of these organ types. 

In summary, the combined analyses of these metabolomics and transcriptomics data have provided new insights into the biosynthetic pathways operating in *D. purpurea* and yielded testable hypotheses about the role of specific enzymes and genes in cardenolide metabolism. As such, this effort has helped to lay a foundation for the further enhancement of this plant species as a production platform for this important class of pharmaceuticals and the potential for its development as a source for new chemical entities addressing these and other medicinal needs.

## 3. Experimental Section

The metabolomes and transcriptomes of medicinal plant species were derived from analysis of up to 20 different plant materials/treatments including major organ types and three developmental stages (young, immature and mature) for most taxa represented. For each species and experimental platform, detailed metadata about plant material, extraction, chromatography, and analytical and computer methodologies, are accessible from the corresponding section of the MPMR database. In brief, plant materials were harvested, quick frozen in liquid nitrogen and stored at -80°C until processing. Samples were extracted for metabolomic determinations by LC/TOF-MS according to Yeo *et al* [73]. (submitted), and in parallel for RNAseq [15]. For LC/TOF-MS analysis, peak detection, integration, and retention-time alignment used automated data processing, based on Waters MarkerLynx software. Export of peak areas was organized by definition of a measured signal based on mass-retention time pairs, individual metabolites were identified (when sufficient information exists) and their relative abundance measured on the basis of their molecular mass (atomic mass units) as reflected by their parent ion generated upon ionization, plus adducts with various salts (i.e. NH_4_^+^ and Na^+^) and, in special cases, possible oligomeric forms (i.e. twice the actual parent ion mass). For analysis by GC-MS, compounds were extracted and analyzed by using targeted assays with known standards [14].

Data in MPMR are stored in a MySQL database. The data are accessed and sent to the client using server-side PHP code. Charts are created using the Javascript charting package Highcharts [74]. The site also uses JQuery [75] and Asynchronous Javascript and XML (AJAX; [76]) for both client-side features (the UI, for example, is based on JQueryUI) and server communications (JQuery's AJAX features are used for searching).

## 4. Conclusions

We describe a novel, publicly accessible database for medicinal plants, and its associated tools for identifying genes and developing metabolic models of specialized pathways. At present, the overwhelming majority of detected metabolites has yet to be annotated or identified. Such annotation is complicated by the existence of multiple isomers of putative key metabolic intermediates. 

Measured metabolite levels provide an important resource for establishing functions of genes responsible for medicinal compound accumulation in plant tissues. This is particularly true in the case, as for most species in MPMR, when transcriptomics data have been gathered from the same samples that were metabolically assayed.

The MPMR database itself can be considered a “live” resource. As methods for identification of additional compounds increase, detailed analyses of the raw data will enable additional progress. The data can inform modeling of the metabolic networks of specialized plant products both by the researchers who deposited the data and by the broader research community. Characterized metabolic pathways and networks are key to the metabolic engineering of natural product composition in the host species, and to develop effective microbial expression platforms for these and structurally-related compounds [3,6,77]. Moreover, combining genes of biosynthetic pathways from different medicinal plants will enable the generation of novel classes of compounds that may not otherwise occur in nature or be readily accessible by chemical synthesis. From a human-health perspective, these data and techniques can enable bioengineering of plants both to produce larger quantities of medicinally-useful compounds as well as to produce new specialized compounds with targeted therapeutic potentials.

Furthermore, modeling of wild plant populations can provide insights into the evolution of plant natural products. Such models will benefit from a more complete understanding of the pathways and the extent of enzyme promiscuity, and will depend on ambitious efforts to isolate intermediates and establish their structures through NMR spectroscopy and/or x-ray crystallography. 
